# Molecular characterization of novel and rare DNA variants in patients with galactosemia

**DOI:** 10.3389/fgene.2023.1266353

**Published:** 2023-11-27

**Authors:** Vasileios Maroulis, Andreas Agathangelidis, Anastasia Skouma, Triantafyllia Sdogou, Manoussos N. Papadakis, Evangelos Papakonstantinou, Panagiotis Girginoudis, Constantinos E. Vorgias, Vassiliki Aleporou, Panagoula Kollia

**Affiliations:** ^1^ Department of Genetics and Biotechnology, Faculty of Biology, School of Physical Sciences, National and Kapodistrian University of Athens, Athens, Greece; ^2^ Department of Newborn Screening, Institute of Child Health, Athens, Greece; ^3^ Neolab SA, Athens, Greece; ^4^ Department of Biochemistry and Molecular Biology, Faculty of Biology, School of Physical Sciences, National and Kapodistrian University of Athens, Athens, Greece

**Keywords:** galactosemia, newborn screening, next-generation sequencing, genetics, molecular analysis, mutation

## Abstract

**Introduction:** Galactosemia is an inherited disorder caused by mutations in the three genes that encode enzymes implicated in galactose catabolism. Currently, the only available treatment for galactosemia is life-long dietary restriction of galactose/lactose, and despite treatment, it might result in long-term complications.

**Methods:** Here, we present five cases of newborn patients with elevated galactose levels, identified in the context of the newborn screening program. Genetic analysis concerned a next generation sequencing (NGS) methodology covering the exons and adjacent splice regions of the *GALT*, *GALK1*, and *GALE* genes.

**Results:** Our approach led to the identification of eight rare nonsynonymous DNA variants. Four of these variants, namely, p.Arg204Gln and p.Met298Ile in *GALT*, p.Arg68Leu in *GALK1*, and p.Ala180Thr in *GALE*, were already recorded in relevant databases, yet their clinical significance is uncertain. The other four variants, namely, p.Phe245Leu in *GALT*, p.Gly193Glu in *GALK1*, and p.Ile266Leu and p.Ala216Thr in the *GALE* gene, were novel. *In silico* analysis of the possible effect of these variants in terms of protein function and stability was performed using a series of bioinformatics tools, followed by visualization of the substituted amino acids within the protein molecule. The analysis revealed a deleterious and/or destabilizing effect for all the variants, supported by multiple tools in each case.

**Discussion:** These results, given the extreme rarity of the variants and the specific phenotype of the respective cases, support a pathogenic effect for each individual variant. Altogether, our study shows that targeted NGS methodologies may offer a time- and cost-effective approach for the genetic investigation of galactosemia and can assist in elucidating the complex genetic background of this disorder.

## 1 Introduction

Hereditary galactosemia is the most common and severe type of disorders related to inborn errors of galactose metabolism. More specifically, the disease is caused by a functional impairment in any of the three enzymes responsible for the conversion of α-D-galactose to glucose-1-phosphate (Holden et al., 2003). These three enzymes constitute the “Leloir pathway” and include galactokinase (GALK, EC:2.7.1.6), galactose-1-phosphate uridyltransferase (GALT, EC 2.7.7.12), and UDP-galactose 4-epimerase (GALE, EC 5.1.3.2). Defects in any of these enzymes are directly linked to the onset of galactosemia, which is inherited in an autosomal recessive manner. GALT deficiency or classic galactosemia (OMIM 230400) caused by mutations in the *GALT* gene emerges during the neonatal period and presents with feeding problems, liver dysfunction, lethargy, bleeding diathesis, growth failure, and sepsis (Berry et al., 2000; Demirbas et al., 2018). Duarte galactosemia is characterized by the presence of a typical *GALT* mutation and the Duarte-2 allele. The latter is associated with the presence of the p.Asn314Asp variant occurring in *cis* configuration with three intronic variants (namely, c.378−27G>C, c.507 + 62G>A, and c.508−24G>A) and a deletion at the *GALT* gene promoter ((c.-119_-116delGTCA). Individuals with Duarte galactosemia exhibit lower GALT activity (15%–35% compared to the germline allele), a moderate increase in blood galactose, and are usually asymptomatic (Demirbas et al., 2018). GALK deficiency or galactosemia II (OMIM 230200) is caused by mutations in the *GALK1* gene, with its main clinical presentation concerning the formation of cataracts due to galactitol accumulation in the lens (Sangiuolo et al., 2004; Demirbas et al., 2018). GALE deficiency (OMIM 230350) is caused by mutations in the *GALE* gene and presents with similar symptoms as those of classic galactosemia, including feeding problems, hypotonia, jaundice, and liver dysfunction (Alano et al., 1998; Demirbas et al., 2018). The prevalence of classic galactosemia is estimated to be 1:40,000–60,000 in Europe and the United States (Berry et al., 2000; Demirbas et al., 2018), while the disease incidence in Greece is estimated to be 1:51,000 (Schulpis et al., 2016). The incidence of GALK deficiency ranges between 1:150,000 and 1:1,000,000 (Sangiuolo et al., 2004), while the frequency of GALE deficiency varies from 1:6,200 to 1:64,800 depending on the ethnic background (Alano et al., 1998). Currently, the only available treatment for all three types of galactosemia is lifelong galactose-/lactose-restricted diet. Despite the application of this treatment, the majority of patients with classic galactosemia have long-term complications including cognitive and/or behavioral problems, speech and motor delay, and premature ovarian insufficiency (Fridovich-Keil et al., 2011). In contrast, the formation of cataracts due to GALK deficiency is usually preventable with diet, while long-term effects have not been observed in the case of GALE deficiency (Cerone and Rios, 2019).

Overall, the increased frequency of galactosemia, its variability in terms of clinical effects, and the lifelong nature of the only available treatment have attracted the interest of researchers in the genetic investigation of the disease. In this context, the integration of galactosemia genetic markers in screening programs was important in the continuous effort for the identification of novel mutations in relevant genes. In Greece, the Institute of Child Health (ICH) is responsible for newborn screening (NS) for the metabolic disorders of phenylketonuria and galactosemia. To date, 363 variants of the *GALT* gene have been recorded in the Associated Regional and University Pathologists (ARUP) database (Calderon et al., 2007), of which 300 variants have been characterized as mutations, with nine of those (3%) being found in Greece (Schulpis et al., 2017). Furthermore, the number of recorded mutations for the *GALK1* and *GALE* genes in ClinVar is 60 and 17, respectively (Landrum et al., 2018).

In the current study, we present five cases of newborn patients with elevated galactose levels identified in the context of the NS program. The application of a targeted re-sequencing approach for the three pathogenic genes, namely, *GALT, GALK1*, and *GALE*, led to the identification of four novel and four rare DNA variants.

## 2 Materials and methods

### 2.1 Study group

Five galactosemia patients (2 with GALT, 2 with GALE, and 1 with GALK deficiency) were identified through the National Newborn Screening (NBS) program. Patients’ follow-up was performed by the clinical department of the Greek Institute of Child Health in compliance with the European Guidelines for Classical Galactosemia (Welling et al., 2017). All procedures were performed in accordance with the ethical standards of the responsible committee on human experimentation in our department and with the Helsinki Declaration of 1975 as revised in 2008.

### 2.2 Study subjects

Patient M1 was a female neonate born to a primigravida mother at full term (after 40 weeks) by cesarean section. Neonatal screening indicated a high level of galactose in the blood (36.4 mg/dL).The patient was admitted to the hospital in good general condition, with jaundice (bilirubin levels of 10.56 mg/dL) and cortical cataract. According to her family history, her maternal grandmother was epileptic for which she received medical treatment. Her GALT enzyme activity was normal (9.5 U/g Hb). She was immediately started on a galactose-free diet and received vitamin D supplementation. Patient M1 maintained normal levels of galactose without psychomotor retardation. During her more recent follow-up, the patient was 4 years old, had no cataract, and did not present with any learning difficulties at the nursery school. Her results of blood biochemical test and ultrasound of abdomen were normal. Finally, the galactose follow-up level on a dried blood spot (DBS) was below 10 mg/dL, while the more recent measurement of galactose was 2.1 mg/dL. She continues the galactose-restricted diet.

Patient T1 was a male neonate born after 39 weeks of gestation via normal delivery. Neonatal screening indicated a high level of galactose in the blood (>50 mg/dL). The patient was admitted to the hospital without any clinical symptoms or cataract. Yet, the patient presented with increased bilirubin levels (16.99 mg/dL) and increased liver function; regarding the latter, both the serum glutamic-oxaloacetic transaminase (SGOT) and serum glutamic pyruvic transferase (SGPT) tests showed elevated levels (>120 IU/dL). GALT enzyme activity was equivocal (3 U/g Hb), and therefore analysis of genes implicated in galactosemia could assist in patient diagnosis. The patient started a galactose-free diet, preserving normal psychomotor development. In his clinical follow-up at the age of 4 years, he presented with normal blood biochemistry, normal eye examination, no psychomotor retardation, and displayed normal learning ability at the nursery school. His weight and height were normal for his age, and he still followed a galactose-restricted diet. The more recent assessment of his galactose level was normal (1,5 mg/dL).

Patient L1 was a female neonate born after 37 weeks of gestation by normal delivery. Neonatal screening indicated a high level of galactose in the blood (64.5 mg/dL). She had only bilateral cataract with no other symptoms and received galactose-free diet and iron supplementation. Her GALT activity was normal, with the GALT measurement at birth being 24 μmol/h/g Hb (normal rate 20–35). Her clinical follow-up remained uneventful with normal galactose levels. The most recent galactose measurement was 2 mg/dL. Eye examination showed that she still has cataract, without any other abnormality. No abnormality was found later during her menstrual period, which started without the need of any hormone supplement. She is now 15 years old, without any difficulties in concentration, yet she has disease phobia and receives regular psychological support. In terms of learning capacity, she is a good student but a very introvert person. She also has anemia and was started on iron supplements. The abdominal ultrasound and bone density test (DXA) results were normal.

Patient D1 was a female neonate born after 39 weeks of gestation by normal delivery. Neonatal screening indicated mildly elevated galactose levels in the blood (15.8 mg/dL), and she was admitted to the hospital to perform the galactosemia diagnostic work-up, where she remained clinically normal and was started on a galactose-free diet. Her GALT activity was normal and psychomotor development was normal on clinical follow-up. Eventually, the special diet was discontinued due to the Duarte mild mutation. She is 4 and a half years old and is currently on a normal diet. Her blood biochemical tests were normal, with no evidence of mental retardation. Galactose levels were normal, with no visual defects. Her growth charts are normal for her age, with the weight being at the 85–97th percentile and the height at the 50th percentile. The most recent measurement showed a galactose level of 1.15 mg/dL.

Patient K1 was a female neonate born after 40 weeks of gestation by normal delivery. Neonatal screening indicated a blood galactose level of 20.4 mg/dL. During hospitalization, the patient displayed normal levels of the relevant liver enzymes without any specific clinical symptoms. GALT activity was estimated at 5.9 U/g Hb, while the ophthalmological examination result was also normal. Nevertheless, she was started on a galactose-free diet, yet for the last 2 years, she has been on a diet with normal galactose levels. She is now 4 and half years old, and besides being on regular galactose diet, the blood biochemical tests were normal, with no evidence of cataract. Despite her good clinical condition, she is hesitant to communicate and needs psychological support. The most recent measurement of galactose was 1.3 mg/dL.

### 2.3 Quantification of galactose levels

Screening procedures have been described in detail previously (Sangiuolo et al., 2004). In brief, total galactose was measured from dried blood spots using enzymatic DELFIA methods with the PerkinElmer GSP analyzer. The cutoff/normal level of total blood galactose was 20 mg/dL, while the measurement of GALT activity was the second-tier test in the diagnostic algorithm.

### 2.4 GALT activity assay

GALT activity was measured using an assay; specifically, GALT in the blood sample catalyzed a reaction between galactose-1-phosphate and uridine diphosphoglucose contained in the assay substrate reagent. In the course of further reactions, nicotinamide adenine dinucleotide phosphate (NADP) also contained in the assay substrate reagent was reduced to NADPH, a fluorescent substance that was measured via excitation at 355 nm and emission detection at 460 nm. Values over 3.5 (U/g Hb) corresponded to normal GALT activity, while values between 2.5 and 3.5 corresponded to equivocal GALT activity. Finally, values below 2.5 corresponded to abnormal GALT activity, which alludes to a pathogenic galactose metabolism. Therefore, the estimation of GALT activity was used as a second-tier test in the galactosemia algorithm.

### 2.5 Genomic analysis

Genomic DNA was extracted from whole blood using the NucleoSpin^®^ Blood mini kit (Macherey-Nagel GmbH, Germany). The quantity of DNA samples was measured using a Qubit^®^ 2.0 fluorometer (Thermo Fisher Scientific, MA, United States). In terms of next-generation sequencing (NGS) analysis, a custom Ion AmpliSeq^TM^ panel was designed, covering the exons, adjacent splice regions, and parts of the 5′ and 3′ UTRs of the *GALT, GALK1*, and *GALE* genes. Library preparation was performed using the Ion AmpliSeq™ Library Kit 2.0 (Thermo Fisher Scientific, MA, United States) according to the manufacturer’s instructions. Emulsion PCR was performed using the Ion OneTouch™ 2 system (Thermo Fisher Scientific, MA, United States), and the libraries were sequenced on an Ion Torrent PGM sequencer (Thermo Fisher Scientific, MA, United States). NGS raw data were analyzed using Torrent Suite Software (Thermo Fisher Scientific, MA, United States), while the visualization of results was performed using the Integrative Genomics Viewer (IGV) (http://software.broadinstitute.org/software/igv/) (Robinson et al., 2011). Genetic analysis of parents toward identifying the configuration of coexisting variants (either in *trans* or in *cis*) was only feasible for patient M1 and was performed with Sanger sequencing.

### 2.6 *In silico* prediction analysis

Evaluation of the possible impact of gene DNA variants on protein function and stability was performed with a series of purpose-built bioinformatics tools. More specifically, the pathogenicity of the variants in terms of protein function was analyzed with the SIFT, Polyphen-2, PROVEAN, Mutation Taster, and CADD tools. In addition, the effect on protein stability was investigated with the Site Directed Mutator (SDM), mCSM, MUpro, and CUPSAT prediction tools. The selection of multiple tools was based on the fact that these tools are among the most widely used and also based on very distinct methodologies, and their performances may vary depending on the setting.

Analysis of the variant effect on protein function with SIFT was based on sequence homology and led to the calculation of a score ranging from 0 to 1. Substitutions with a score of <0.05 are characterized as deleterious, while those with a score of ≥0.05 are considered tolerated (Kumar et al., 2009). Polyphen-2 uses sequence-based and structure-based features to classify variants. The Polyphen-2 score represents the probability of a substitution to be damaging; scores between 0 and 0.15 are associated with benign variants, while scores between 0.15 and 1 are assigned to possibly damaging variants (Adzhubei et al., 2010). PROVEAN performs an alignment of the query sequence with related protein sequences and calculates an alignment score. If the score is equal or below a certain threshold (by default 2.5), the variant is predicted to be deleterious; otherwise, it is considered neutral (Choi and Chan, 2015). Mutation Taster classifies variants by incorporating data from common polymorphisms and known mutations extracted from relevant databases and the literature, as well as conservation data from the alignment of nucleotide and amino acid sequences with the respective sequences of ten other species. The tool provides a prediction together with its probability; a score close to 1 indicates a high probability (Schwarz et al., 2010). Finally, CADD compares real against simulated variants and integrates multiple annotations into one metric, the C-score. The C-score is strongly correlated with pathogenicity, with a value of 10 indicating that the variant is among the 10% of most deleterious nucleotide changes in the human genome. For the identification of deleterious variants, the default cutoff is set between 10 and 20 (Rentzsch et al., 2019). For these reasons, the cutoff for the detection of relevant variants in the present study was set to 15.

SDM uses environment-specific substitution tables (ESSTs) to calculate the difference in stability between the wild-type and mutated proteins. ESSTs are calculated for each family of proteins and provide the probability that an amino acid substitution may occur in a specific local structural environment that is defined by main-chain conformational angles and secondary structures, relative solvent accessibility, and hydrogen bonding patterns (Pandurangan et al., 2017). mCSM uses a graph-based approach where the amino acid environment is defined by atoms within a certain distance. Distance patterns are then extracted from the pairwise distances of the atoms as feature vectors called mCSM signatures. mCSM signatures and vectors that represent the difference between the reference and replacement amino acids are then used to train predictive models for protein stability and protein interactions (Pires et al., 2014). MUpro uses support-vector machines (SVMs) trained in a dataset of 1,615 single-site mutations from 42 proteins extracted from the thermodynamic database for proteins and mutants ProTherm (Gromiha et al., 2000) to predict the stability changes caused by mutations (Cheng et al., 2006). CUPSAT predicts changes in protein stability due to point mutations using a prediction model based on atom potentials and torsion angle potentials derived from a set of 4,024 protein structures. The tool also incorporates information regarding the secondary structure and solvent accessibility (Parthiban et al., 2006). Visualization of the three-dimensional structure of the protein and the respective residues was performed using Visual Molecular Dynamics (Humphrey et al., 1996).

### 2.7 *In silico* structural analysis

*In silico* protein structural analysis was employed for the assessment of the impact of the identified variants on protein structure. The applied methodology can be categorized in three main levels: (i) primary sequence collection, (ii) modeling of the three-dimensional (3D) protein structures, and (iii) 3D protein structure comparison.

The first step concerned the collection of sequences of the *GALT*, *GALE*, and *GALK1* genes with or without the identified variants (germline/wildtype form); given the inability to assess whether concurrent variants in the same gene were present in *cis* or *trans* configuration, all possible combinations of variants were taken into account. In particular, the GALT sequence dataset included the germline sequence as well as sequences carrying the individual Arg204Gln, Met298Ile, Asn314Asp, and Phe245Leu variants as well as the combinations of Arg204Gln/Met298Ile and Asn314Asp/Phe245Leu. In the case of the *GALE* gene, the germline sequence as well as sequences carrying the Ala216Thr, Ala180Thr, and Ile266Leu variants were included in the analysis. Regarding the GALK1 gene, sequence collection involved the germline counterpart as well as those sequences carrying the Arg68Leu and Gly193Glu variants and their combination.

This set of 15 protein amino acid sequences were transformed to 3D protein models, using the SWISS-MODEL protein structure prediction tool (https://swissmodel.expasy.org/), which represents a fully automated protein structure homology-modeling analyzer. This process resulted in a set of protein models in PDB format. Structural alignment was performed between the germline form of each gene and all its respective variants using the PDBeFold tool, and the structural similarity score that corresponds to the overall root mean standard deviation (RMSD) was computed. The RMSD metric is well-established for the comparison of protein structures, while it is also a reliable indicator of variability when applied to very similar proteins. Finally, differences were visualized using the PyMOL tool.

## 3 Results

In total, five cases of patients presenting with clinical and biochemical indices of galactosemia were examined. Overall, the genetic investigation of the *GALT, GALK1*, and *GALE* genes led to the identification of eight DNA variants, which were either novel or had an unknown effect; detailed information regarding these variants is given in Table 1.

**TABLE 1:** Detailed characterization of identified DNA variants in the *GALT, GALK1*, and *GALE* genes.

Case ID	Sex	Allele 1				Allele 2				Allele 3			
		Gene	Variant at protein level	Variant at cDNA level	Population frequency	Gene	Variant at protein level	Variant at cDNA level	Population frequency	Gene	Variant at protein level	Variant at cDNA level	Population frequency
D1	F	*GALT*	p.Phe245Leu	c.735C>G	Absent from population databases	*GALT*	p.Asn314Asp	Duarte-2 (c.-119_-116delGTCA, c.940A>G)	4.6% gnomAD	-	-	-	-
K1	F	*GALT*	p.Arg204Gln	c.611G>A	0.005% gnomAD	*GALT*	p.Met298Ile	c.894G>A	0.005% ALPHA	-	-	-	-
M1	F	*GALE*	p.Ile266Leu	c.796A>C	Absent from population databases	*GALE*	p.Ala180Thr	c.538G>A	0.003% gnomAD	-	-	-	-
				rs758252537				rs756590751					
T1	M	*GALE*	p.Ala216Thr	c.646G>A	Absent from population databases	*GALE*	p.Ala216Thr	c.646G>A	Absent from population databases	-	-	-	-
L1	F	*GALK1*	p.Gly193Glu	c.578G>A	Absent from population databases	*GALK1*	p.Arg68Leu	c.203G>T	0.001% gnomAD	*GALT*	p.Asn314Asp	Duarte-2 (c.-119_-116delGTCA, c.940A>G)	4.6% gnomAD

Concerning classical galactosemia (GALT deficiency), three cases (namely, D1, K1, and L1) were found to carry five variants in the *GALT* gene, four of which were unique (the Duarte-2 variant was identified in cases D1 and L1). The analysis of the individual *GALT* variants showed that three of them were either novel (p.Phe245Leu) or rare (p.Arg204Gln and p.Met298Ile). Variant p.Arg204Gln was recorded in dbSNP and gnomAD, while it was also recorded in ClinVar as of uncertain significance. The other previously described *GALT* variant (p.Met298Ile) was identified in two siblings and two other independent cases with classic galactosemia (Schulpis et al., 2017) and was recorded in the dbSNP and ALPHA databases; no record of this variant was found in ClinVar.

Three novel variants were also identified in the *GALE* (namely, p.Ile266Leu and p.Ala216Thr) and *GALK1* genes (p.Gly193Glu). These variants were not present in any of the dbSNP, gnomAD, and ClinVar databases. In contrast, variants p.Ala180Thr and p.Arg68Leu identified in the *GALE* and *GALK1* genes, respectively, were previously recorded in dbSNP and/or gnomAD. Moreover, the *GALK1* p.Arg68Leu variant was recorded in ClinVar as of uncertain significance.

The genotypes of the individual cases of the present study were as follows: (i) *GALT:* Phe245Leu/Duarte-2 either in *cis* or *trans* configuration (case D1), (ii) *GALT:* p.Arg204Gln/p.Met298Ile either in *cis* or *trans* configuration (case K1), (iii) *GALE:* Ile266Leu/Ala180Thr in *trans* configuration (case M1), (iv) *GALE:* Ala216Thr/Ala216Thr with the possibility of a deletion in *trans* configuration (case T1), and (v) *GALK1:* Gly193Glu/Arg68Leu either in *cis* or *trans* configuration and *GALT:* Duarte-2/Nl (case L1).

### 3.1 Analysis of variants in the *GALT* gene

**FIGURE 1:**
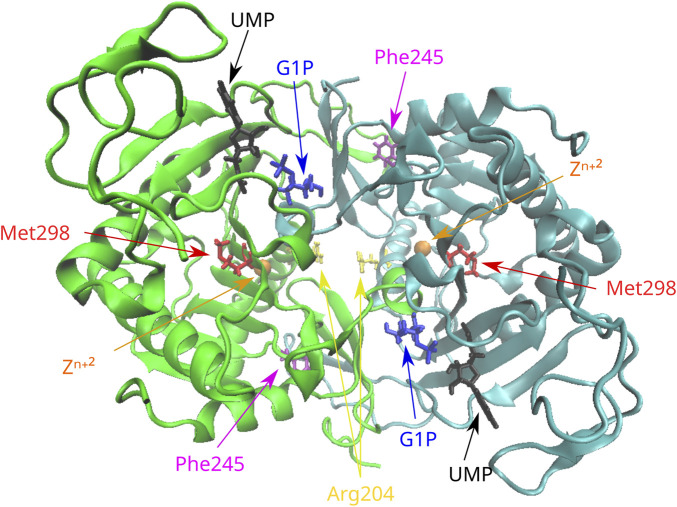
Position of the missense variants in the 3D structure of the GALT dimer. Phe245 is located in a loop of the molecule in proximity with residues of the other subunit. Arg204 is located in an alpha helix in the exterior of the molecule. Met298 is located in a beta sheet in the interior of the molecule. G1P, glucose-1-phosphate; UMP, uridine monophosphate.

The novel amino acid change of Phe at position 245 for Leu (Phe245Leu) corresponded to an important physicochemical alteration of the R-amino acid group. *In silico* analysis led to its characterization as pathogenic (Table 2) by all applied functional prediction tools, while two out of the four stability prediction tools predicted a destabilizing effect, with CUPSAT also predicting unfavorable torsion angles. The GALT enzyme is an obligate dimer also requiring zinc for its function. Phe245 is located at a loop of the amino acid chain away from the active site or the zinc-binding site of the GALT enzyme (Figure 1), but in close proximity (<5 angstroms) with three amino acid residues (at positions 117, 118, and 119) located within a beta sheet of the other subunit. Hence, the p.Phe245Leu variant may affect subunit interaction, although such a role has not been extensively documented (McCorvie et al., 2016). The analysis of the rare amino acid change p.Arg204Gln showed contradictory results (Table 2), with Mutation Taster and CADD characterizing it as deleterious and the rest of the tools as neutral, even though this substitution led to a clear change in terms of polarity. Regarding protein stability, three out of the four stability prediction tools predicted a destabilizing effect, with only CUPSAT predicting favorable torsion angles. Arg204 is located away from the active site or the zinc binding site of the enzyme (Figure 1), within an alpha helix in the exterior of the molecule with no indications that this region may be implicated in subunit interaction.

**TABLE 2:** *In silico* data analysis for the functional and stability effects of each variant at the protein level.

Gene	Variant at protein level	SIFT prediction | score	Polyphen-2 prediction | score	PROVEAN prediction |	Mutation taster prediction	CADD Phred score	SDM prediction	mCSM prediction	MUpro prediction	CUPSAT prediction	
										Stability	Torsion
*GALT*	p.Phe245Leu	Damaging	Possibly damaging	Deleterious	Disease causing	24,9	Increased stability	Destabilizing	Decrease stability	Stabilizing	Unfavorable
		000.002	0.763	−4.23							
*GALT*	p.Arg204Gln	Tolerated	Benign	Neutral	Disease causing	21,7	Increased stability	Destabilizing	Decrease stability	Destabilizing	Favorable
		000.321	0.007	−0.97							
*GALT*	p.Met298Ile	Damaging	Probably damaging	Deleterious	Disease causing	29,3	Increased stability	Destabilizing	Decrease stability	Stabilizing	Unfavorable
		000.001	1.0	−3.76							
*GALE*	p.Ile266Leu	Tolerated	Benign	Neutral	Disease causing	22,6	Reduced stability	Destabilizing	Decrease stability	Stabilizing	Favorable
		000.071	0.003	−1.37							
*GALE*	p.Ala180Thr	Damaging	Probably damaging	Neutral	Disease causing	25,9	Reduced stability	Destabilizing	Decrease stability	Destabilizing	Unfavorable
		000.021	0.96	−0.35							
*GALE*	p.Ala216Thr	Damaging	Probably damaging	Deleterious	Disease causing	28	Reduced stability	Destabilizing	Decrease stability	Stabilizing	Unfavorable
		0.0	1.0	−3.70							
*GALK1*	p.Gly193Glu	Damaging	Probably damaging	Deleterious	Disease causing	23,7	Reduced stability	Destabilizing	Decrease stability	Destabilizing	Unfavorable
		000.002	0.999	−6.58							
*GALK1*	p.Arg68Leu	Tolerated	Benign	Neutral	Polymorphism	15,41	Reduced stability	Destabilizing	Decrease stability	Destabilizing	No change
		000.705	0.009	−1.48							

The variant p.Met298Ile was considered deleterious after the *in silico* analysis by all functional prediction tools, even though both amino acids involved in the substitution are large and hydrophobic. Two out of the four stability prediction tools predicted a destabilizing effect, with CUPSAT also predicting unfavorable torsion angles. Met298 is located within a beta sheet in the interior of the molecule (Figure 1) and is not associated with either the active and zinc-binding sites of the enzyme or with subunit interaction.

### 3.2 Analysis of variants in the *GALE* gene

**FIGURE 2:**
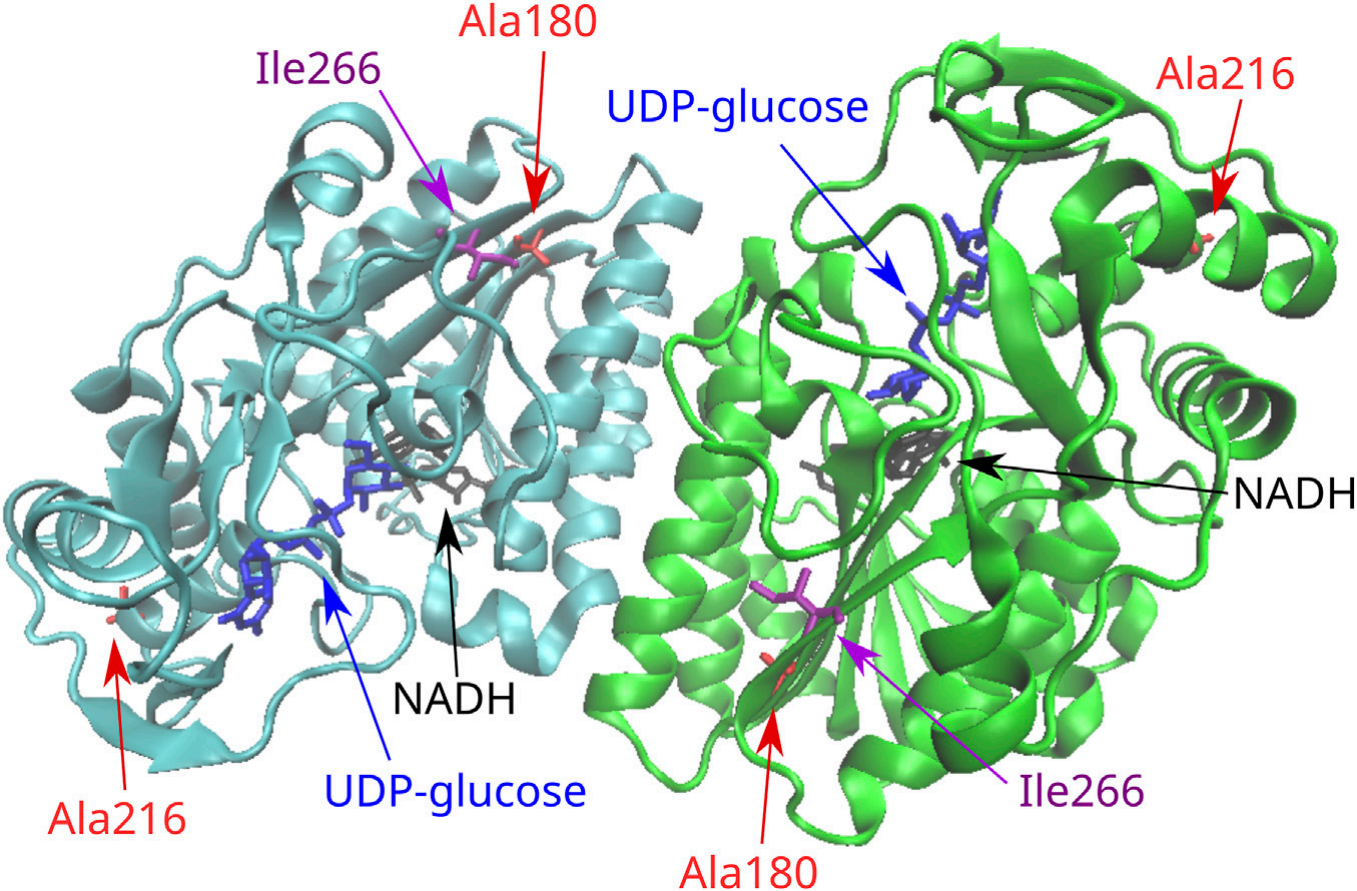
Position of the missense variants in the 3D structure of the GALE dimer. Ala216 is located in one end of an alpha helix in the C-terminal domain of the molecule. Ile266 is located in the C-terminal domain in a beta strand that forms a parallel beta sheet with six other beta strands from the N-terminal domain. This seven-stranded beta sheet is responsible for the positioning of NAD+. Ala180 is located in a beta strand of the N-terminal domain that forms the same seven-stranded beta sheet.

The novel variant p.Ile266Leu, which was located at exon 10, did not concern a significant change in the polarity of the amino acid R-group since both implicated amino acids are aliphatic. In terms of functional prediction, only Mutation Taster and CADD characterized this variant as deleterious. Three out of the four stability prediction tools predicted a destabilizing effect, while only CUPSAT predicted a stabilizing effect as well as favorable torsion angles (Table 2). The GALE enzyme also functions as a dimer, and Ile266 is located at the C-terminal domain of the subunits within a beta strand. This beta strand forms a parallel beta sheet with six other beta strands from the N-terminal domain (Figure 2), which is responsible for the positioning of NAD+. Yet Ile266 is not proximal to the active site nor is implicated in subunit interactions (Thoden et al., 2000).

The rare variant p.Ala180Thr resulted in a significant change from a hydrophobic to a polar R-group. As probably expected, all function prediction tools employed in the *in silico* analysis characterized the variant as deleterious, with the exception of PROVEAN. Likewise, all stability prediction tools predicted a destabilizing effect with unfavorable torsion angles. Ala180 is located at a beta strand of the N-terminal domain of the enzyme, which forms the same seven-stranded beta sheet responsible for the positioning of NAD+, although again the residue is located away from the active site and is not implicated in subunit interactions.

The novel *GALE* variant p.Ala216Thr leads to a change of the hydrophobic R-group of alanine to the polar R-group of threonine. *In silico* analysis showed pathogenic predictions by all functional prediction tools. In line with this, all stability prediction tools reported a destabilizing effect. The only exception was CUPSAT, which predicted an overall stabilizing effect but unfavorable torsion angles. Ala216 is located at the C-terminal domain at one end of an alpha helix, which is not proximal to the active site of GALE. Of relevance, the other end of the helix is proximal enough to form interactions with the substrate. Again, there are no indications that this residue is implicated in subunit interactions.

### 3.3 Analysis of variants in the *GALK1* gene

**FIGURE 3:**
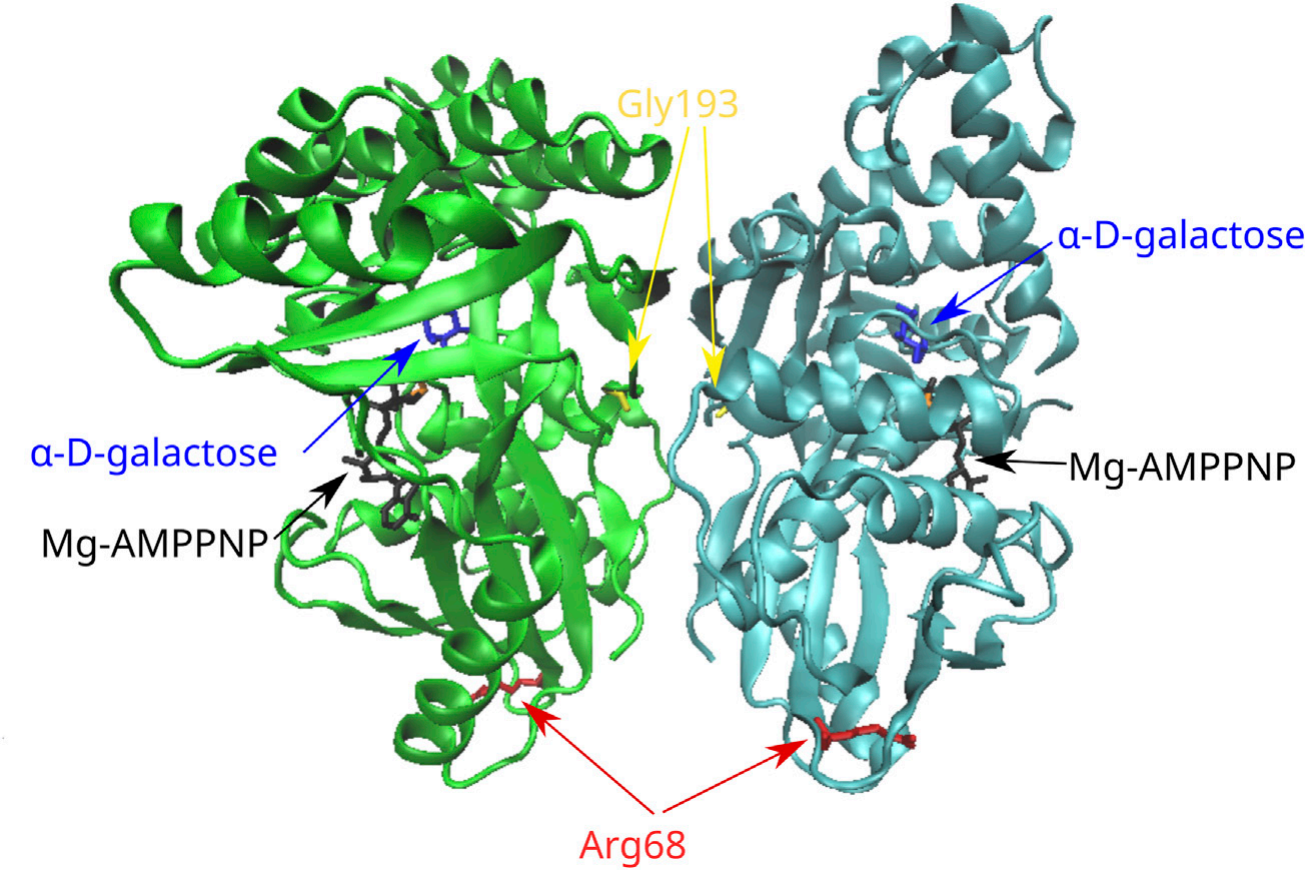
Position of the missense variants in the 3D structure of the GALK dimer. Gly193 is located in a loop of the molecule that belongs to the subunit–subunit interface. Arg68 is located in a six-stranded beta sheet in the N-terminal region of the molecule. AMPPNP, adenylyl imidodiphosphate (an ATP analog).

Τhe novel variant p.Gly193Glu concerns an amino acid change from the polar R-chain of glycine to the negatively charged R-chain of glutamate; a prediction of pathogenicity was supported by the *in silico* analysis with all aforementioned functional prediction tools. Furthermore, a destabilizing effect with unfavorable torsion angles was also supported by all stability prediction tools (Table 2). It has been suggested that the GALK enzyme functions as a dimer (Thoden et al., 2005); although Gly193 is not proximal to its active site, it is located at a loop of the molecule (Figure 3) that belongs to the subunit–subunit interface.

In the case of the rare variant p.Arg68Leu, the positively charged R-chain of arginine is replaced by the hydrophobic R-group of leucine. All *in silico* functional prediction tools classified this variant as neutral; in contrast, all stability prediction tools showed a destabilizing effect, with CUPSAT predicting no change in torsion angles. Arg68 is located at the N-terminal region of the molecule within a six-stranded beta sheet that is distal to the active site. Furthermore, there are no indications for the implication of this residue in subunit interactions.

### 3.4 Assessment of variant impact on protein structure

**FIGURE 4:**
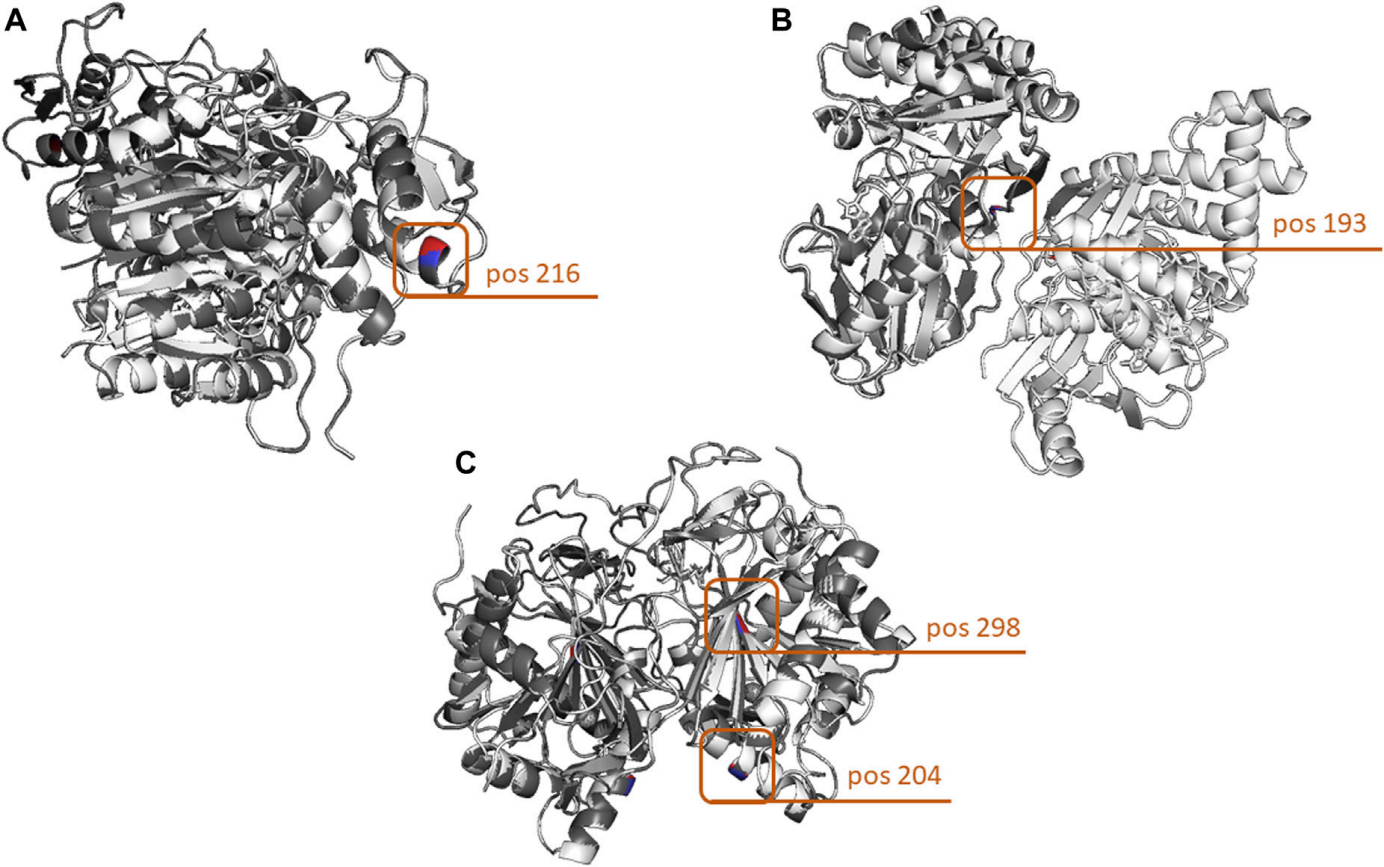
Three-dimensional comparison of the *GALE, GALK, and GALT* protein structures encoded by the germline versions of the genes and the variants with the highest structural impact. (A) In the case of the *GALE* gene, the highest structural impact was evidenced by the change of Ala to Thr at amino acid position 216. **(B)** In terms of protein structure, the most impactful *GALK1* variant leads to the change of Gly to Glu at amino acid position 193. **(C)** Finally, the strongest effect in the structure of the GALT protein was evidenced by the combination of two variants leading to the Arg204Gln and Met298Ile amino acid changes. Protein structures were loaded and aligned using the PyMOL tool. The amino acid chains of the germline forms of the proteins are depicted in white, whereas those carrying the variants are depicted in gray. In terms of the amino acid positions carrying the variants, the germline-encoded amino acid is depicted in red, and that encoded by the variant in blue.

Given the fact that our genetic analysis in the *GALE*, *GALK1*, and *GALT* genes revealed the presence of either individual variants or, at most, combinations of two variants in each patient, the overall impact on the protein structure was, as expected, rather minor. However, different findings were evident depending on the gene; in the case of the *GALE* gene, the effect of the Ala180Thr and Ile266Leu variants was quite minor (range of overall RMSD: 0.02–0.05), while in contrast, the effect of the Ala216Thr variant had an almost 10-fold stronger impact (Figure 4A). The results were largely different when focusing on the *GALK1* gene variants; more specifically, both the Arg68Leu and Gly193Glu variants displayed a considerable effect on protein structure with an RMSD value of 0.44 and 0.443 (Figure 4B), respectively. However, these variants were shown to neutralize each other since their combination showed a significantly lower overall RMSD value (0.08) than that of the protein structure encoded by the germline *GALK1* gene. Finally, the Arg204Gln variant of the *GALT* gene displayed a significantly stronger impact on the protein structure than the Met298Ile (overall RMSD of 0.134 versus only 0.012). As expected, their combination had an overall RMSD value similar to that of the former variant (0.135), indicating its impact (Figure 4C). In regard to the Asn314Asp and Phe245Leu variants, they both displayed a significant effect on the protein structure of the *GALT* gene, with an overall RMSD value of 0.134 in both cases. Yet, as in the case of the *GALK1* variants, the combination of variants (Asn314Asp/Phe245Leu) led to an overall protein structure that was very similar to the germline (overall RMSD of 0.007). The overall RMSD distance values for individual variants as well as variant combinations for the *GALE*, *GALK1*, and *GALT* genes are given in Table 3.

**TABLE 3:** Assessment of comparison and 3D alignment of protein structures between the germline configuration and all possible variants of the *GALT*, *GALE*, and *GALK1* genes.

Gene	Variant	Overall RMSD distance
*GALT*	Arg204Gln	0.134
	Met298Ile	0.012
	Arg204Gln/Met298Ile	0.135
	Asn314Asp	0.134
	Phe245Leu	0.134
	Asn314Asp/Phe245Leu	0.007
*GALE*	Ala216Thr	0.048
	Ala180Thr	0.005
	Ile266Leu	0.002
*GALK1*	Arg68Leu/Gly193Glu	0.08
	Arg68Leu	0.44
	Gly193Glu	0.443

## 4 Discussion

To date, a large number of mutations have been recorded in the *GALT* gene, with the respective numbers being considerably smaller for the *GALK1* and *GALE* genes (Cooper et al., 1998). The existence of this largely heterogeneous genetic background suggests that it may affect, at least in part, the phenotypic variability of the disease. Within this complex genetic background, there are some mutations that predominate; more specifically, p.Gln188Arg is the most frequent *GALT* mutation and together with p.Lys285Asn, p.Ser135Leu, and p.Leu195Pro was reported to account for up to more than 70% of the mutated *GALT* alleles in certain population studies (Tyfield et al., 1999; Boutron et al., 2012). In terms of phenotypic impact, the p.Gln188Arg, p.Lys285Asn, and p.Leu195Pro *GALT* mutations have been associated with a more severe phenotype, whereas the p.Ile32Asn, p.Thr138Met, p.Thr350Ala, p.Arg259Trp, and p.Ala330Val mutations have been associated with a milder one (Tyfield et al., 1999). The very common Duarte-2 *GALT* variant, characterized by a population frequency of 4%, has a mild effect with homozygotes, displaying 50% of the normal enzyme activity (Elsas et al., 1994). In regard to *GALE* mutations, the p.Val94Met variant is associated with the more severe, generalized form of the disease (Timson, 2006). Overall, reports on the association between genotype and phenotype are relatively scarce; of interest, patients with identical genotypes and biochemical features may demonstrate high variability in terms of the clinical phenotype (Elsas and Lai, 1998; Welsink-Karssies et al., 2020).

In the present study, we analyzed five cases with galactosemia diagnosed in the context of the Greek National NS program for galactosemia. Genetic analysis of the galactosemia genes, namely, *GALT*, *GALE*, and *GALK1*, led to the identification of four novel and four rare variants of uncertain significance. Two of the cases, namely, M1 and K1, were heterozygotes for either a novel and a rare variant or two rare variants in the *GALE* and *GALT* genes, respectively. Case T1 was a homozygote for a novel variant in the *GALE* gene, whereas case D1 was a heterozygote for a novel variant and an established mild mutation in the *GALT* gene (Duarte-2). Finally, case L1 was a heterozygote for a novel and a rare variant in the *GALK1* gene and also a heterozygote for the mild mutation Duarte-2 in the *GALT* gene. Genetic analysis of the parents to confirm the *trans/cis* status of concurrent variants was feasible only in the case of patient M1, which showed that the implicated variants were present in *trans* configuration*.* Therefore, for the rest of the cases, the configuration of coexisting variants was not assessed. The small size of our cohort was due to a strict selection of cases with particular clinical and biochemical features. Hence, the results of this study, even though largely informative, might not fully capture the genetic background of the Greek galactosemia population and its mutational spectrum.

According to the *in silico* variant analysis, all or at least the majority of functional prediction tools assigned a deleterious effect to *GALT* variants p.Phe245Leu and p.Met298Ile, *GALE* variants p.Ala180Thr and p.Ala216Thr, as well as the *GALK1* variant p.Gly193Glu. For the variants p.Arg204Gln and p.Ile266Leu of the *GALT* and *GALE* genes, respectively, predictions were contradictory; more specifically, Mutation Taster and CADD predicted a deleterious effect for both variants, contrasting the prediction of the rest of the tools that characterized them as neutral. Finally, the *GALK1* variant p.Arg68Leu was predicted to be neutral by all tools.

Regarding protein stability, *GALK1* variants p.Arg68Leu and p.Gly193Glu as well as the *GALE* variant p.Ala180Thr were predicted to have a destabilizing effect by all bioinformatics tools, while the *GALE* variants p.Ala216Thr and p.Ile266Leu and the *GALT* variant p.Arg204Gln were predicted to have a destabilizing effect by the majority of the tools. For the rare and novel *GALT* variants p.Met298Ile and p.Phe245Leu, respectively, protein stability predictions were contradictory. Notably, all aforementioned variants were predicted to have at least a deleterious or a destabilizing effect by the majority of the bioinformatics tools.

Finally, the analysis of the impact of the identified *GALE*, *GALK1*, and *GALT* gene variants on protein structure led to quite variant results depending on the gene and the variants. Of importance, the results from the *in silico* 3D protein structure analysis showed a good level of concordance with those of the aforementioned analyses on protein function and stability, at least in most cases, strengthening the relevance of our findings. Concerning the *GALE* gene, the strongest impact on protein structure was evident for the Ala216Thr variant followed by the Ala180Thr variant; both of these variants were also shown to significantly affect the function and stability of the protein. In terms of the *GALK1* gene, the Gly193Glu and Arg68Leu variants were shown to be the most impactful at the level of protein structure; the former was also characterized by a significant effect on protein function and stability, whereas the latter was characterized by a protein destabilizing effect. A similar scenario was observed for the Phe245Leu and Arg204Gln variants of the *GALT* gene, both being characterized by a strong impact on protein structure; the former was also associated with a strong impact on protein function, while the latter displayed a significant impact on protein stability. The only case of disagreement between the *in silico* analyses on protein function/stability and structure concerned the *GALT* Asn314Asp and the Met298Ile variants; the Asn314Asp variant was characterized by a strong impact on protein structure yet displayed no effect on function and stability, while the Met298Ile variant was associated only with an effect on protein function without affecting the structure and stability of the molecule.

Patient D1 harbored the novel *GALT* p.Phe245Leu variant together with the mild Duarte-2 variant. The mild biochemical and clinical features of this case are consistent with the identification of the Duarte-2 variant, although it cannot be excluded that variant p.Phe245Leu may also have a mild effect. The latter was predicted to be pathogenic by all functional prediction tools, while two out of four stability prediction tools showed a destabilizing effect. Due to its position in the 3D structure of the enzyme, there is a possibility that the Phe245 residue is implicated in the subunit interaction of the GALT dimer. Interestingly, an amino acid change at the adjacent amino acid position (p.Phe244Ser) was previously described in a case with classic galactosemia in combination with a common *GALT* mutation (Viggiano et al., 2015), indicating that this region may be important for the function of the molecule. According to the ACMG guidelines (Richards et al., 2015), p.Phe245Leu is classified as a variant of uncertain significance (PM2, PP3, and PP4).

Patient K1 carried the rare p.Met298Ile and p.Arg204Gln variants in the *GALT* gene, which were related to a milder increase in galactose levels compared to the other cases, suggesting that either one of these variants or both of them may have a mild effect. Variant Met298Ile was predicted as pathogenic by all functional prediction tools, while two out of four of the stability prediction tools showed a destabilizing effect. This variant has already been described in cases with classic galactosemia of Greek origin, where in combination with known *GALT* mutations it resulted in a typical galactosemia phenotype (Schulpis et al., 2017). Functional predictions in the case of the variant p.Arg204Gln were contradictory, yet the majority of the stability prediction tools predicted a destabilizing effect. Of relevance, a different variant at the same position (p.Arg204Pro) was identified at the gnomAD database at a very low prevalence (0.005%, 7/140230 alleles), which was characterized as pathogenic (Zekanowski et al., 1999). There is no evidence that either of these variants is implicated either in the function of the enzyme, due to their positioning away from the active site, or in its subunit interactions. Finally, both the p.Met298Ile and p.Arg204Gln variants have a very low population frequency (0.005% in ALPHA and gnomAD databases for both variants), another feature compatible with their probable pathogenic effect. According to the ACMG guidelines, both the p.Met298Ile (PP3, PP4, PP5, and PS4 as moderate due to the rarity of the variant) and p.Arg204Gln (PP4) variants are classified as variants of uncertain significance.

Patient M1 was a compound heterozygote for the novel p.Ile266Leu and the rare p.Ala180Thr *GALE* variants. The biochemical and clinical features of this case were typical, including elevated galactose and bilirubin levels in the blood along with cataract development. Function predictions concerning p.Ile266Leu were contradictory, while the majority of stability prediction tools supported a destabilizing effect. Regarding the p.Ala180Thr variant, the majority of the function predictions supported a deleterious effect, whereas all the stability predictions supported a destabilizing effect. There is no evidence that either of these residues may be implicated in the function of the active site or the subunit interactions of the enzyme. The highly specific phenotype in combination with the *in silico* predictions, the novelty of p.Ile266Leu, and the very low population frequency of p.Ala180Thr (0.003% in gnomAD) support a causative effect for the combination of these variants. According to the ACMG guidelines, p.Ile266Leu (PM2, PP4) and p.Ala180Thr (PP3, PP4) are classified as variants of uncertain significance.

Patient T1 was homozygous for the novel p.Ala216Thr variant in the *GALE* gene. The patient presented with typical biochemical and clinical features. The variant p.Ala216Thr was predicted to be pathogenic by all the functional prediction tools, whereas the majority of the stability tools predicted a destabilizing effect. In terms of impact, there was not sufficient evidence that this residue has any implication in the active site or the subunit interactions of the enzyme. Interestingly, another variant at the same amino acid position (p.Ala216Val) in compound heterozygosity with another variant of uncertain clinical significance was described in a case with elevated galactose level (37.6 mg/dL) suspected of GALE deficiency. Of importance, GALE activity in the patients’ erythrocytes was reduced to only 0.5% of the control mean (Li et al., 2014). Thus, the *in silico* predictions in combination with the absence of the p.Ala216Thr variant from population databases support the probable pathogenicity of this variant. According to ACMG guidelines, p.Ala216Thr is classified as a variant of uncertain significance (PM2, PP3, and PP4).

Finally, patient L1 who presented with typical biochemical and clinical features of GALK deficiency was a compound heterozygote for the novel variant p.Gly193Glu and the rare variant p.Arg68Leu, both in the *GALK1* gene. Variant p.Gly193Glu was predicted to have a deleterious effect as well as a destabilizing effect by all prediction tools, while p.Arg68Leu was predicted to be neutral by all functional prediction tools, yet destabilizing by all stability prediction tools. Regarding their relative position in the 3D structure of the enzyme, there was no evidence that Arg68 is implicated either in the active site or subunit interactions of the enzyme, even though Gly193 is located at a loop of the molecule that belongs to the interface of the enzyme subunits. These features in combination with the highly specific and typical phenotype, the novelty of p.Gly193Glu, and the low population frequency of p.Arg68Leu (0.001% in gnomAD) support a causative effect for the combination of these variants. Interestingly enough, this patient also harbored the known, mild Duarte-2 mutation in the *GALT* gene. This highlights the notion that the genetic basis of galactosemia disorders can be rather complex, underlining the importance of genetic testing in the characterization of these cases. According to ACMG guidelines, p.Gly193Glu (PM2, PP3, and PP4) and p.Arg68Leu (PP4) are classified as variants of uncertain significance.

Galactosemia testing was incorporated in the Greek NS programs to provide early diagnosis of the disorder and prevent any dangerous neonatal complications through a galactose-restricted diet. Most of the cases in our study presented with a typical biochemical and clinical galactosemia phenotype, and the *in silico* analysis of the identified variants together with their absence or very low frequency in population databases supports a pathogenic effect for these variants. In the cases with a milder biochemical and clinical phenotype, this could be attributed to either the presence of a known mild variant (case D1) or to a possible mild effect of the newly identified variants (case K1). Our results show that the application of NGS methods offers a time- and cost-effective approach for the comprehensive genetic investigation of all three genes implicated in galactosemia. This can, in turn, lead to the identification of novel and rare variants as well as assist in the elucidation of the complex genetic background of this disorder.

## Data Availability

The datasets for this article are not publicly available due to concerns regarding patient anonymity. Requests to access the datasets should be directed to the corresponding author.
